# A hybrid decision support model to discover informative knowledge in diagnosing acute appendicitis

**DOI:** 10.1186/1472-6947-12-17

**Published:** 2012-03-13

**Authors:** Chang Sik Son, Byoung Kuk Jang, Suk Tae Seo, Min Soo Kim, Yoon Nyun Kim

**Affiliations:** 1 Department of Medical Informatics, School of Medicine, Keimyung University, 2800 Dalgubeoldaero, Dalseo-Gu, Daegu, Republic of Korea; 2 Department of Internal Medicine, School of Medicine, Keimyung University, 2800 Dalgubeoldaero, Dalseo-Gu, Daegu, Republic of Korea; 3 Biomedical Information Technology Center, School of Medicine, Keimyung University, 2800 Dalgubeoldaero, Dalseo-Gu, Daegu, Republic of Korea

**Keywords:** Hybrid decision support model, Acute appendicitis, Knowledge discovery, Decision tree, Logistic regression analysis

## Abstract

**Background:**

The aim of this study is to develop a simple and reliable hybrid decision support model by combining statistical analysis and decision tree algorithms to ensure high accuracy of early diagnosis in patients with suspected acute appendicitis and to identify useful decision rules.

**Methods:**

We enrolled 326 patients who attended an emergency medical center complaining mainly of acute abdominal pain. Statistical analysis approaches were used as a feature selection process in the design of decision support models, including the Chi-square test, Fisher's exact test, the Mann-Whitney *U*-test (*p *< 0.01), and Wald forward logistic regression (entry and removal criteria of 0.01 and 0.05, or 0.05 and 0.10, respectively). The final decision support models were constructed using the C5.0 decision tree algorithm of Clementine 12.0 after pre-processing.

**Results:**

Of 55 variables, two subsets were found to be indispensable for early diagnostic knowledge discovery in acute appendicitis. The two subsets were as follows: (1) lymphocytes, urine glucose, total bilirubin, total amylase, chloride, red blood cell, neutrophils, eosinophils, white blood cell, complaints, basophils, glucose, monocytes, activated partial thromboplastin time, urine ketone, and direct bilirubin in the univariate analysis-based model; and (2) neutrophils, complaints, total bilirubin, urine glucose, and lipase in the multivariate analysis-based model. The experimental results showed that the model with univariate analysis (80.2%, 82.4%, 78.3%, 76.8%, 83.5%, and 80.3%) outperformed models using multivariate analysis (71.6%, 69.3%, 73.7%, 69.7%, 73.3%, and 71.5% with entry and removal criteria of 0.01 and 0.05; 73.5%, 66.0%, 80.0%, 74.3%, 72.9%, and 73.0% with entry and removal criteria of 0.05 and 0.10) in terms of accuracy, sensitivity, specificity, positive predictive value, negative predictive value, and area under ROC curve, during a 10-fold cross validation. A statistically significant difference was detected in the pairwise comparison of ROC curves (*p *< 0.01, 95% CI, 3.13-14.5; *p *< 0.05, 95% CI, 1.54-13.1). The larger induced decision model was more effective for identifying acute appendicitis in patients with acute abdominal pain, whereas the smaller induced decision tree was less accurate with the test data.

**Conclusions:**

The decision model developed in this study can be applied as an aid in the initial decision making of clinicians to increase vigilance in cases of suspected acute appendicitis.

## Background

Acute appendicitis is a common disease in emergency abdominal surgery with a lifetime occurrence of approximately 7% and perforation rates of 17-20% [1-3]. The decision to explore a patient with suspected acute appendicitis is based mainly on disease history and physical findings, but the clinical presentation is seldom typical [4]. Unfortunately, some patients with acute appendicitis are not diagnosed until the occurrence of peritonitis or other severe complications while their surgeons are waiting for more evidence of acute appendicitis. These patients have a higher mortality and morbidity than patients who are diagnosed in a timely manner [5]. Thus, a timely and accurate diagnosis of acute appendicitis is important for avoiding unnecessary diagnostic procedures and for identifying appropriate therapeutic measures and clinical management strategies. However, finding meaningful factors and identifying their relationships is difficult due to the numerous parameters that are routinely available, such as patient history and laboratory data, etc.

Computer-aided diagnosis of acute abdominal pain has challenged researchers for over 40 years. Since the pioneering work of de Dombal et al. [6], several studies have aimed to support the diagnosis of acute appendicitis on the basis of grading medical history, clinical symptoms, and signs [7-9]. Eberhart [10] reported a comparison of appendicitis diagnosis versus non-specific abdominal pain using three different neural network paradigms: back propagation (BP), binary adaptive resonance theory (ART-1), and fuzzy resonance (Fuzzy-ART). Pesonen [11] compared the predictive performance of four different neural network algorithms in the diagnosis of acute appendicitis with different parameter groups, i.e., ART-1, self-organizing maps (SOM), learning vector quantization (LVQ), and BP. It was found that supervised learning algorithms (LVQ and BP) performed better than unsupervised learning algorithms (ART-1 and SOM) in medical decision making problems. Prabhudesai [12] evaluated artificial neural networks (ANNs) for the diagnosis of appendicitis in patients presenting with acute right iliac fossa (RIF) pain and compared ANN performance with assessments made by experienced clinicians and the Alvarado score [13]. The ability of ANNs to accurately exclude the diagnosis of appendicitis in patients without true appendicitis was significantly better than clinical performance and an Alvarado score ≥ 6. All the neural network algorithms provided good performances in the diagnosis of acute appendicitis, but they had the following drawbacks: time-consuming depending on the size of training data, a black-box structure lacking transparency in the knowledge generated, and the inability to explain the decisions that were made.

Several other studies of acute abdominal pain and acute appendicitis have been performed, including decision tree models. The performance of these models ranged from 43% to 95% [5,14-16]. Ting [5] modified the Alvarado scoring system (ASS) with a decision tree technique and constructed a convenient and accurate decision support model that consisted of RLQ tenderness, the Alvarado score, migrating pain, and a neutrophil count > 75% for acute appendicitis diagnosis and timing of laparotomy. Gaga [14] introduced the data representation formalism ID+, which was derived from Quinlan's ID3 algorithm, to facilitate the modeling of dependencies between attributes or attribute values, with multiple values per attribute. They used this method to demonstrate a medical knowledge acquisition application for abdominal pain in children. Ohmann [15] evaluated the performance of seven knowledge acquisition techniques, i.e., Bayes independence and rule induction techniques, ID3, NewId, PRISM, CN2, C4.5, and ITRULE. No overall differences in accuracy were observed, except with NewId, which was less accurate compared with the other algorithms. None of the algorithms produced an overall accuracy of > 50%. Zorman [16] addressed the problem of separating acute appendicitis from other diseases causing acute abdominal pain with an improved decision tree approach based on the dynamic discretization of continuous attributes. This method was used to investigate the predictive performance of different decision trees with three prospective databases: the COMAC-BME-European Community Concerted Action on Objective Medical Decision Making in Patients with Acute Abdominal Pain project, the German MEDWIS project A70 "Expert system for acute abdominal pain," and the COPERNICUS program no. 555 project.

Most of these studies have focused on the issues, the discriminatory power of decision support models, or the decision rules derived from different decision tree algorithms without performing a statistical comparison of the significance of their results. In this study, we present a hybrid decision support model that combines statistical analysis and decision tree approaches to discover significant rules and provide high accuracy, early diagnosis for patients with suspected acute appendicitis.

## Methods

### Data

After obtaining the Institutional Review Board (IRB) approval (no. 11-275) from Keimyung University Dongsan Hospital, we retrospectively collected the medical records of all patients attending the emergency medical center complaining mainly of acute abdominal pain between July 2006 and June 2007. Only complete medical records with no missing clinical parameters were included, i.e., age, gender, chief complaints, and clinical laboratory findings, such as urinalysis, common blood cell and differential counts, serum electrolytes, routine admission, etc. To analyze the chief complaints, we split the abdomen areas into eight distinct regions (Table 1) based on four abdominal quadrants [14], i.e., the right upper quadrant (RUQ), right lower quadrant (RLQ), left upper quadrant (LUQ), and left lower quadrant (LLQ). Patients diagnosed with complaints other than appendicitis were excluded, such as acute cholecystitis or diverticulitis, appendectomy incidental to another surgical procedure, previous use of antibiotics for chronic appendicitis, and appendectomy for chronic abdominal pain. The eligibility for study group (n = 152) was defined according to the International Classification of Diseases-10 (ICD-10) codes: K35.0 (acute appendicitis with generalized peritonitis), K35.1 (acute appendicitis with peritoneal abscess), and K35.9 (acute appendicitis without generalized peritonitis). Discharged patients (n = 174) admitted to the emergency medical center who complained mainly of acute abdominal pain were defined as the control group. All data collected were reconfirmed by gastroenterologists.

**Table 1:** Comparison of patient characteristics (age, gender, chief complaints, and urinalysis) for acute appendicitis and discharged patients (n = 326)

Characteristics	AA* (n = 152)	Control** (n = 174)	*p *value
Age, yrs^†^	36.57 ± 21.31	43.05 ± 20.86	0.003^††^

Gender			0.021
Male	77 (50.7%)	66 (37.9%)	
Female	75 (49.3%)	108 (62.1%)	

Chief complaints			0.000^††^
abdominal	76 (50.0%)	109 (62.6%)	
left upper quadrant (LUQ)	0 (0.0%)	1 (0.6%)	
periumbilical area	4 (2.6%)	10 (5.7%)	
right lower quadrant (RLQ)	60 (39.5%)	16 (9.2%)	
left lower quadrant (LLQ)	0 (0.0%)	5 (2.9%)	
lower abdominal	5 (3.3%)	18 (10.3%)	
right upper quadrant (RUQ)	6 (3.9%)	15 (8.6%)	
upper abdominal	1 (0.7%)	0 (0.0%)	

Urinalysis			
Color			0.407
amber	10 (6.6%)	6 (3.4%)	
brown	1 (0.7%)	1 (0.6%)	
straw	140 (92.1%)	167 (96.0%)	
yellow	1 (0.7%)	0 (0.0%)	
S.G.^†^	1.02 ± 0.01	1.02 ± 0.01	0.106
pH^†^	6.61 ± 0.93	6.43 ± 0.92	0.086
Albumin			0.412
negative	127 (83.6%)	151 (86.8%)	
positive	25 (16.4%)	23 (13.2%)	
Glucose			0.000^††^
negative	116 (76.3%)	170 (97.7%)	
positive	36 (23.7%)	4 (2.3%)	
Ketone			0.000^††^
negative	98 (64.5%)	149 (85.6%)	
positive	54 (35.5%)	25 (14.4%)	
O.B.			0.578
negative	104 (68.4%)	114 (65.5%)	
positive	48 (31.6%)	60 (34.5%)	
Urobilinogen, E.U./dL^†^	0.52 ± 1.49	0.22 ± 0.54	0.020
Bilirubin			0.983
negative	139 (91.4%)	159 (91.4%)	
positive	13 (8.6%)	15 (8.6%)	
Nitrite			0.689
negative	150 (98.7%)	170 (97.7%)	
positive	2 (1.3%)	4 (2.3%)	
WBC1			0.345
negative	99 (65.1%)	122 (70.1%)	
positive	53 (34.9%)	52 (29.9%)	
RBC			0.972
negative	53 (34.9%)	61 (35.1%)	
positive	99 (65.1%)	113 (64.9%)	
WBC2			0.936
negative	11 (7.2%)	13 (7.5%)	
positive	141 (92.8%)	161 (92.5%)	
Ep. Cell			0.303
negative	30 (19.7%)	26 (14.9%)	
positive	122 (80.3%)	148 (85.1%)	
Cast			0.284
negative	151 (99.3%)	174 (100.0%)	
positive	1 (0.7%)	0 (0.0%)	
Other			0.184
negative	147 (96.7%)	172 (98.9%)	
positive	5 (3.3%)	2 (1.1%)	
Crystal			0.383
negative	151 (99.3%)	171 (98.3%)	
positive	1 (0.7%)	3 (1.7%)	

* acute appendicitis; ** discharged patients; ^† ^Mean ± SD; ^†† ^*p *< 0.01

### Statistical analysis and decision tree model

Statistical analysis was performed using SPSS 12.0 for Windows (SPSS Inc., Chicago, IL, USA). Univariate correlations between clinical or laboratory features were evaluated using the Chi-square test or Fisher's exact test, which are appropriate for categorical data, and using the Student *t*-test or Mann-Whitney *U*-test with continuous variables, after checking for normality using the Kolmogorov-Smirnov test. A two-tailed *p *< 0.01 was selected as the level of statistical significance. In the multivariate analysis, the Wald forward logistic regression model, with entry and removal criteria of 0.01 and 0.05, or 0.05 and 0.10, respectively, was used to identify independent predictors of acute appendicitis. Modeling results were expressed as the odds ratios (OR) with 95% confidence intervals (95% CI). The Hosmer-Lemeshow test (*H*) was used to assess the fit of the models, which divides subjects into deciles based on their predicted probabilities before computing Chi-square values from the observed and expected frequencies [17-20].

After the feature selection process, Quinlan's C5.0 decision tree algorithm [21,22] was used to design the final decision support models. This approach provides a very simple representation of accumulated knowledge and it also facilitates the derivation of an explanation for the decision, which is essential in medical applications. The model selects the best decision node that separates the different classes from the empirical data [23]. The main induction loop of the decision tree is as follows [5]: i) assume A as the possible "best" decision attribute for the next node; ii) assign A as the decision attribute for the node; iii) for each value of A, create a new descendent of the node; iv) count the entropies of the training examples to the leaf nodes; and v) stop searching for new leaf nodes if training examples are well-classified, or continue the new leaf nodes if they are not well-classified. The decision tree model used in this study was built with C5.0 component using the default experimental parameters of Clementine version 12.0 (SPSS Inc., Chicago, IL, USA).

### Structure of the clinical decision support model and its evaluation

**Figure 1:**
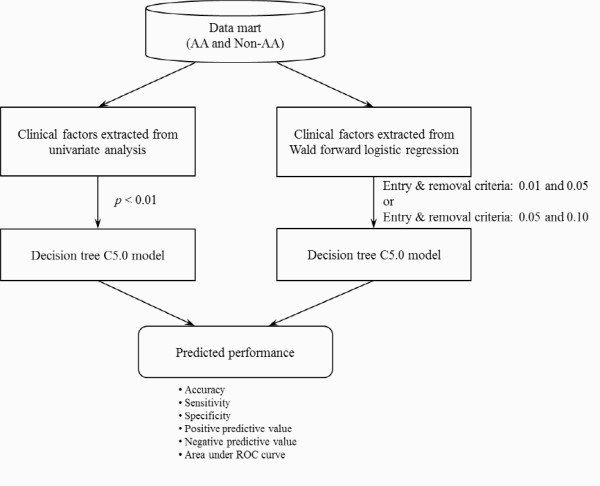
**Scheme of the decision support models**.

Figure 1 shows the scheme of the decision support models, which were based on statistical tests (i.e., univariate analysis, *p *< 0.01) and the Wald forward logistic regression (entry and removal criteria of 0.01 and 0.05 or 0.05 and 0.10), for diagnosis of acute appendicitis. We used 10-fold cross validation experiments to provide an unbiased estimate of the generalization error. The full dataset was randomly divided into 10 subsets: nine subsets were used for training (90%), while the remaining subset was used for testing (10%). The process was then repeated 10 times. The performance of the models was evaluated using six standard measures: accuracy (ACC), sensitivity (SENS), specificity (SPEC), positive predictive value (PPV), negative predictive value (NPV), and the area under the ROC curve (AUC). We also made a pairwise comparison [24,25] between the ROC curves of the models to test for statistically significant differences.

## Results

Of the 326 patients enrolled in this study, 152 (46.6%) had acute appendicitis, while 174 (53.4%) were discharged. Significant differences were observed in terms of age (*p *< 0.01), complaints (*p *< 0.001), urine glucose (*p *< 0.001), and urine ketone (*p *< 0.001), among the acute appendicitis patients (mean age, 36.57 years) and the discharged patients (mean age, 43.05 years). Abdominal and RLQ pains were the most common complaints presented in the emergency medical center (Table 1).

In terms of blood test findings, white blood cell (*p *< 0.001), red blood cell (*p *< 0.01), neutrophils (*p *< 0.001), glucose (*p *< 0.01), total bilirubin (*p *< 0.001), direct bilirubin (*p *< 0.001), and activated partial thromboplastin time (*p *< 0.01) were significantly or slightly higher in patients with acute appendicitis, whereas lymphocytes (*p *< 0.001), monocytes (*p *< 0.001), eosinophils (*p *< 0.001), basophils (*p *< 0.001), large unstained cells (*p *< 0.001), sodium (*p *< 0.001), chloride (*p *< 0.001), lipase (*p *< 0.001), and total amylase (*p *< 0.001) were significantly higher in discharged patients. The remaining variables could not be used to differentiate acute appendicitis from the discharged patients (Table 2).

**Table 2:** Comparison of patient characteristics (CBC & Differential Count, Serum Electrolytes, Routine Admission, etc.) for acute appendicitis and discharged patients (n = 326)

Characteristics	AA* (n = 152)	Control** (n = 174)	*p *value
CBC & Differential Count^†^			
WBC, × 10^3^/μL	13.47 ± 4.90	9.47 ± 3.91	0.000^††^
RBC, × 10^3^/μL	4.50 ± 0.55	4.29 ± 0.59	0.001^††^
HGB, g/dL	13.51 ± 1.83	13.08 ± 1.83	0.037
HCT,%	39.03 ± 4.92	37.93 ± 5.23	0.027
MCV, fl	86.96 ± 5.54	88.58 ± 5.69	0.010
MCH, pg	30.16 ± 2.24	30.62 ± 2.32	0.074
MCHC, g/dL	34.73 ± 1.33	34.59 ± 1.34	0.343
PLT, × 10^3^/μL	268.69 ± 81.29	262.43 ± 78.51	0.480
NEUT,%	81.48 ± 8.62	69.28 ± 14.14	0.000^††^
LYMP,%	12.51 ± 6.95	22.75 ± 12.49	0.000^††^
MONO,%	4.08 ± 1.91	4.67 ± 1.78	0.000^††^
EOS,%	1.30 ± 0.91	2.14 ± 1.70	0.000^††^
BASO,%	0.37 ± 0.27	0.49 ± 0.37	0.000^††^
LUC,%	1.20 ± 0.73	1.73 ± 0.99	0.000^††^
MPV, fl	7.72 ± 0.68	7.76 ± 0.73	0.633

Serum Electrolyte^†^			
Na, mmol/L	143.11 ± 2.83	144.01 ± 3.35	0.000^††^
K, mmol/L	4.19 ± 0.44	4.21 ± 0.42	0.705
Cl, mmol/L	106.12 ± 3.25	107.25 ± 3.83	0.000^††^

Routine Admission^†^			
Calcium (T), mg/dL	9.11 ± 0.52	9.10 ± 0.52	0.874
Inorganic Phosphorus, mg/dL	3.36 ± 0.82	3.43 ± 0.80	0.490
Glucose, mg/dL	127.39 ± 36.23	116.61 ± 40.64	0.001^††^
BUN, mg/dL	13.15 ± 7.92	13.86 ± 6.44	0.376
Creatinine, mg/dL	0.88 ± 0.25	0.94 ± 0.51	0.229
Cholesterol (T), mg/dL	169.19 ± 37.91	174.44 ± 38.84	0.219
Protein (T), g/dL	7.21 ± 0.63	7.18 ± 0.57	0.650
Albumin, g/dL	4.23 ± 0.37	4.20 ± 0.36	0.404
Bilirubin (T), mg/dL	1.24 ± 0.66	0.90 ± 0.48	0.000^††^
Bilirubin (D), mg/dL	0.36 ± 0.16	0.26 ± 0.17	0.000^††^
ALP, U/L	97.61 ± 70.85	88.05 ± 52.93	0.174
AST, U/L	25.70 ± 12.84	31.01 ± 27.80	0.025
ALT, U/L	22.97 ± 20.90	24.70 ± 19.75	0.444

APTT^†^, s	29.92 ± 4.78	29.43 ± 12.01	0.007^††^
PT^†^, s	1.03 ± 0.12	1.01 ± 0.13	0.028
Lipase^†^, U/L	24.14 ± 7.70	31.70 ± 16.89	0.000^††^
Amylase (T)^†^, U/L	46.86 ± 27.77	55.04 ± 26.90	0.000^††^

* acute appendicitis; ** discharge patients; ^† ^Mean ± SD; ^†† ^*p *< 0.01

In the multivariate analysis, independent risk factors were identified using Wald forward logistic regression, to define entry and removal criteria of 0.01 and 0.05, or 0.05 and 0.10, respectively. Regardless of the criteria used, the independent risk factors provided the same results using the two logistic models. We included six variables in the final logistic regression that were independently associated with acute appendicitis: complaints, urine glucose, white blood cell, neutrophils, total bilirubin, and lipase (Table 3). These variables were tested by linear regression analysis to evaluate multicollinearity among the predictors. The data did not violate the multicollinearity assumption. The tolerance of each independent variable was greater than 0.616. The variance inflation factor (VIF) values of the variables ranged from 1.005 to 1.624. The ACC, SENS, SPEC, PPV, and NPV, were 79.8%, 76.3%, 82.8%, 79.5%, and 80.0%, respectively. The AUC of the models was 79.5% (95% CI, 74.7-83.8), indicating fair discriminatory power. The goodness-of-fit (*H*) statistic indicated that the models were well calibrated (*p *= 0.838).

**Table 3:** Multivariate analysis of predictors of acute appendicitis (entry and removal criteria of 0.01 and 0.05, or 0.05 and 0.10)

Variables	Coefficient (β)	Standard error	OR (95% CI)	*p *value	*H *statistic*
Complaints**				0.000	
LUQ	-19.774	40192.97	-	1.000	
PA^†^	-0.725	0.754	0.484 (0.111-2.121)	0.336	
RLQ	1.838	0.397	6.281 (2.883-13.687)	0.000	
LLQ	-18.848	17365.29	-	0.999	
Lower abd.	-1.215	0.697	0.297 (0.076-1.163)	0.081	
RUQ	-0.047	0.784	0.954 (0.205-4.433)	0.952	
Upper abd.	19.292	40192.97	2.390E8 (-)	1.000	0.838
Urine glucose (positive)	2.537	0.644	12.636 (3.575-44.658)	0.000	
WBC	0.116	0.043	1.123 (1.033-1.221)	0.007	
NEUT	0.057	0.017	1.059 (1.023-1.095)	0.001	
Bilirubin (T)	0.795	0.268	2.213 (1.308-3.746)	0.003	
Lipase	-0.042	0.016	0.958 (0.928-0.989)	0.009	
Intercept	-6.035	1.290	-	0.000	

R^2 ^= 0.428; n = 326

* Hosmer-Lemeshow goodness-of-fit (*H*) statistic

** abdominal pain as reference category

^† ^periumbilical area

### Decision support model based on multivariate analysis

**Figure 2:**
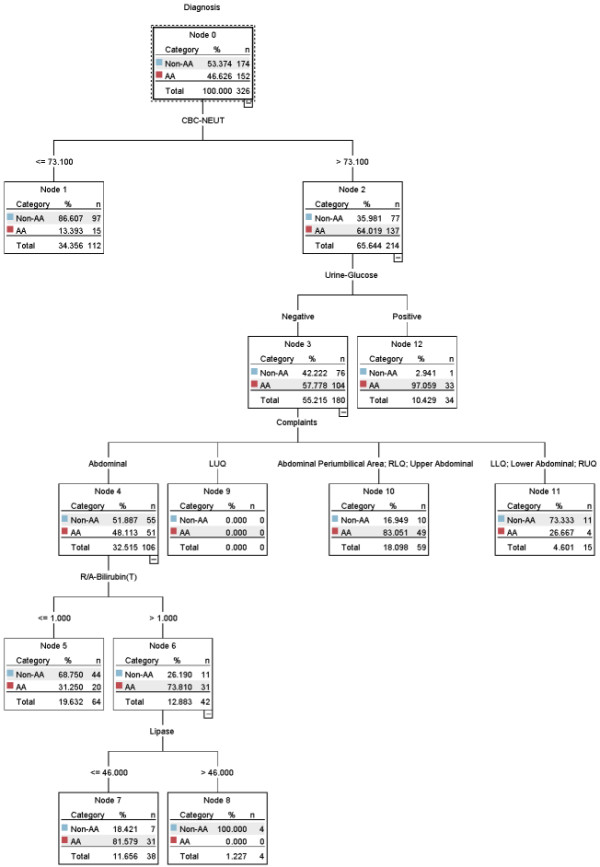
**Decision support model based on Wald logistic regression (entry and removal of 0.01 and 0.05, or 0.05 and 0.10)**.

Five of the six variables (Table 3) were selected by the C5.0 decision tree model and their importance was defined in the following order: neutrophils, complaints, total bilirubin, urine glucose, and lipase. The cut-off points were determined using the C5.0 decision tree algorithm and the criteria for dichotomizing the continuous variables were all statistically significant (*p *< 0.05) except for LUQ pain (OR, 0.732; 95% CI, 0.014-37.307; *p *= 0.876). The results are summarized in Table 4. The decision support model is shown in Figure 2 and eight decision rules were generated from the full dataset. Seven decision rules (in Figure 2, leaf nodes 1, 5, 7, 8, 10, 11, and 12) were statistically significant, excluding leaf node LUQ (node 9). Three rules were associated with acute appendicitis as follows: 1) neutrophils > 73.1% and urine glucose is positive (*p *< 0.01); 2) neutrophils > 73.1% and urine glucose is negative and periumbilical area pain, or upper abdominal pain, or RLQ pain (*p *< 0.001); 3) neutrophils > 73.1% and urine glucose is negative and abdominal pain, and total bilirubin > 1.0 mg/dL, and lipase ≤ 46 U/L (*p *< 0.05). The ACC, SENS, SPEC, PPV, NPV, and AUC measures were 82.5%, 74.3%, 89.7%, 86.3%, 80.0%, and 82.0% (95% CI, 77.4-86.0), respectively.

**Table 4:** Statistical significance of cut-off points determined using the C5.0 decision tree algorithm (for multivariate analysis)

Variables	OR (95% CI)	*p *value
**Level 0 (root node)**		
NEUT ≤ 73.1% or > 73.1%	11.506 (6.244-21.202)	0.000
**Level 1**		
Urine glucose (negative)	0.041 (0.006-0.310)	0.002
Urine glucose (positive)	24.115 (3.227-180.216)	0.002
Level 2*		
Abdominal pain	2.722 (1.445-5.125)	0.002
LUQ pain	0.732 (0.014-37.307)	0.876
PA or RLQ or Upper abdominal pain	5.880 (2.727-12.681)	0.000
LLQ or Lower abdominal or RUQ pain	4.231 (1.292-13.855)	0.017
**Level 3**		
Bilirubin (T) ≤ 1.0 mg/dL or > 1.0 mg/dL	6.200 (2.604-14.762)	0.000
**Level 4**		
Lipase ≤ 46 U/L or > 46 U/L	37.800 (1.829-781.085)	0.019

* LUQ, left upper quadrant; PA, periumbilical area; RLQ, right lower quadrant; LLQ, left lower quadrant; RUQ, right upper quadrant

### Decision support model based on univariate analysis

**Figure 3:**
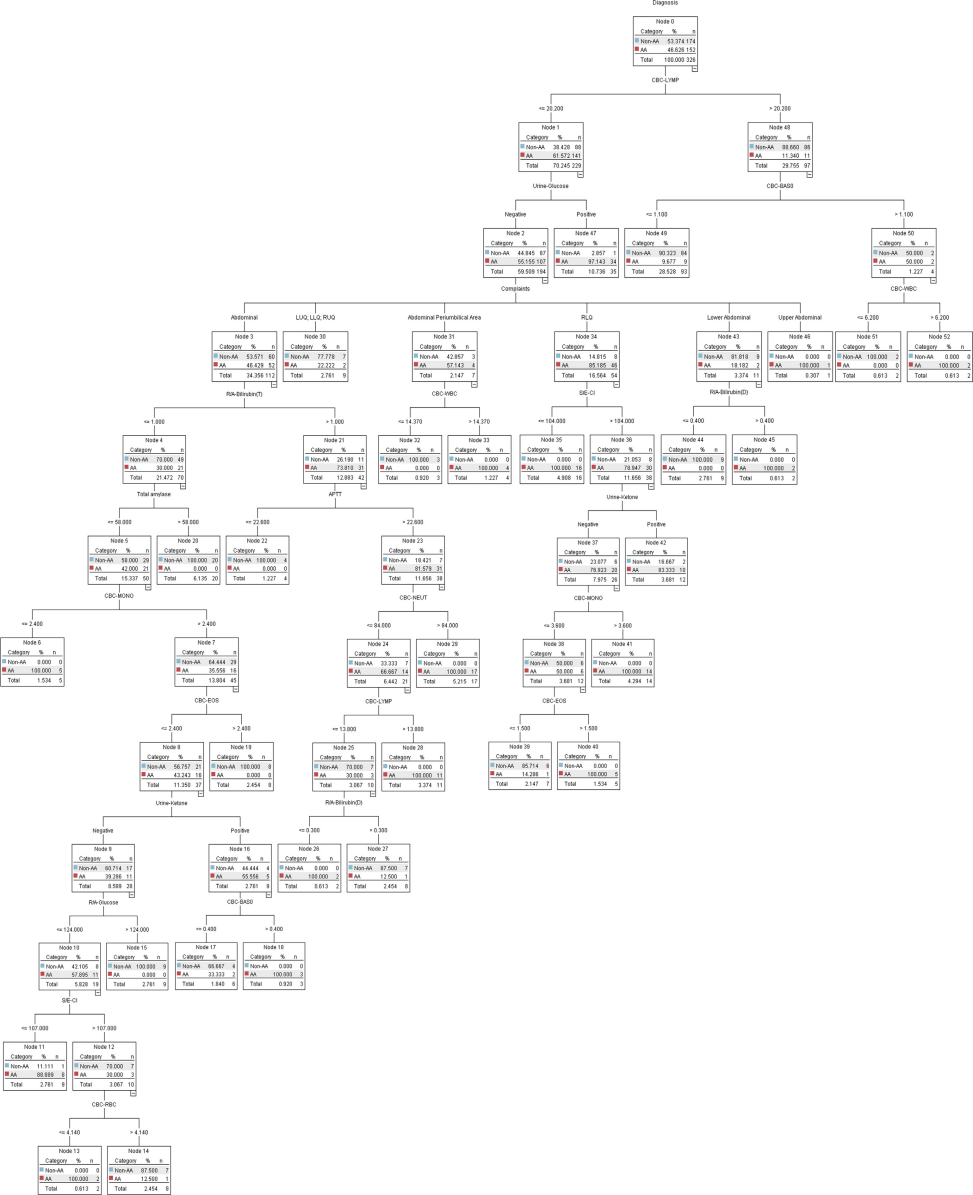
**Decision support model based on univariate analysis**.

Sixteen of the 20 variables with *p *< 0.01 (Tables 1 and 2) were selected by the C5.0 decision tree algorithm and their importance was defined in the following order: lymphocytes, urine glucose, total bilirubin, total amylase, chloride, red blood cell, neutrophils, eosinophils, white blood cell, complaints, basophils, glucose, monocytes, activated partial thromboplastin time, urine ketone, and direct bilirubin. The criteria for the selected cut-off points are summarized in Table 5. The decision support model for the diagnosis of acute appendicitis is shown in Figure 3 and its performance was 93.9%, 89.5%, 97.7%, 97.1%, 91.4%, and 93.6% (95% CI, 90.4-96.0). We generated 29 decision rules, i.e., 16 for acute appendicitis and 13 for the control group. Thirteen decision rules (in Figure 3: leaf nodes 6, 11, 15, 20, 22, 28, 39, 40, 41, 44, 45, 47, and 49) were statistically significant. Seven rules were associated with acute appendicitis as follows: 1) lymphocytes ≤ 20.2% and urine glucose is positive (*p *< 0.01); 2) lymphocytes ≤ 20.2% and urine glucose is negative and lower abdominal pain and direct bilirubin > 0.4 mg/dL (*p *< 0.05); 3) lymphocytes ≤ 20.2% and urine glucose is negative and RLQ pain and chloride > 104 mmol/L and urine ketone is negative and monocytes > 3.6% (*p *< 0.05); 4) lymphocytes ≤ 20.2% and urine glucose is negative and RLQ pain and chloride > 104 mmol/L and urine ketone is negative and monocytes ≤ 3.6% and eosinophils > 1.5% (*p *< 0.05); 5) lymphocytes ≤ 20.2% and urine glucose is negative and abdominal pain and total bilirubin ≤ 1.0 mg/dL and total amylase ≤ 58 U and monocytes ≤ 2.4% (*p *< 0.05); 6) lymphocytes ≤ 20.2% and urine glucose is negative and abdominal pain and total bilirubin > 1.0 mg/dL and activated partial thromboplastin time > 22.6 s and neutrophils ≤ 84% and lymphocytes > 13.8% (*p *< 0.05); 7) lymphocytes ≤ 20.2% and urine glucose is negative and abdominal pain and total bilirubin ≤ 1.0 mg/dL and total amylase ≤ 58 U and monocytes > 2.4% and eosinophils ≤ 2.4% and urine ketone is negative and glucose ≤ 124 mg/dL and chloride ≤ 107 mmol/L (*p *< 0.05).

**Table 5:** Statistical significance of cut-off points determined using the C5.0 decision tree algorithm (for univariate analysis)

Variables	OR (95% CI)	*p *value
**Level 0 (root node)**		
LYMP ≤ 20.2% or > 20.2%	12.527 (6.335-24.770)	0.000
**Level 1**		
Urine glucose (negative)	0.036 (0.005-0.270)	0.001
Urine glucose (positive)	27.645 (3.709-206.043)	0.001
BASO ≤ 1.1%	9.333 (1.169-74.489)	0.035
BASO > 1.1%	0.107 (0.013-0.8555)	0.035
Level 2*		
Abdominal pain	4.487 (2.556-7.878)	0.000
LUQ or LLQ or RUQ pain	4.594 (0.929-22.712)	0.062
APA pain	1.087 (0.237-4.994)	0.914
RLQ pain	7.447 (3.274-16.938)	0.000
Lower abdominal pain	6.058 (1.273-28.826)	0.024
Upper abdominal pain	2.465 (0.099-61.267)	0.582
WBC ≤ 6.2 × 10^3^/μL or > 6.2 × 10^3^/μL	25.000 (0.341-1831.738)	0.142
**Level 3**		
Bilirubin (T) ≤ 1.0 mg/dL or > 1.0 mg/dL	6.576 (2.791-15.493)	0.000
WBC ≤ 14.37 × 10^3^/μL or > 14.37 × 10^3^/μL	63.000 (0.982-4042.374)	0.051
Cl ≤ 104 mmol/L	9.197 (0.499-169.573)	0.136
Cl > 104 mmol/L	0.109 (0.006-2.005)	0.136
Bilirubin (D) ≤ 0.4 mg/dL or > 0.4 mg/dL	95.000 (1.482-6088.126)	0.032
**Level 4**		
Amylase (T) ≤ 58 U/L	0.033 (0.002-0.584)	0.020
Amylase (T) > 58 U/L	29.881 (1.1711-521.765)	0.020
APTT ≤ 22.6 s or > 22.6 s	37.800 (1.829-781.085)	0.019
Urine ketone (negative)	0.667 (0.113-3.919)	0.654
Urine ketone (positive)	1.500 (0.255-8.817)	0.654
**Level 5**		
MONO ≤ 2.4% or > 2.4%	19.667 (1.022-378.446)	0.048
NEUT ≤ 84%	0.055 (0.003-1.051)	0.054
NEUT > 84%	18.103 (0.951-344.559)	0.054
MONO ≤ 3.6% or > 3.6%	29.000 (1.413-595.209)	0.029
**Level 6**		
EOS ≤ 2.4%	0.077 (0.004-1.426)	0.085
EOS > 2.4%	13.047 (0.701-242.739)	0.085
LYMP ≤ 13.8% or > 13.8%	49.286 (2.214-1097.056)	0.014
EOS ≤ 1.5% or > 1.5%	47.667 (1.597-1422.784)	0.026
**Level 7**		
Urine ketone (negative or positive)	1.932 (0.423-8.814)	0.395
Bilirubin (D) ≤ 0.3 mg/dL or > 0.3 mg/dL	25.000 (0.750-832.997)	0.072
**Level 8**		
Glucose ≤ 124 mg/dL or > 124 mg/dL	25.706 (1.307-505.514)	0.033
BASO ≤ 0.4% or > 0.4%	12.600 (0.446-356.388)	0.137
**Level 9**		
Cl ≤ 107 mmol/L or > 107 mmol/L	18.667 (1.563-222.937)	0.021
**Level 10**		
RBC ≤ 4.14 × 10^3^/μL or > 4.14 × 10^3^/μL	25.000 (0.750-832.997)	0.072

* LUQ, left upper quadrant; LLQ, left lower quadrant; RUQ, right upper quadrant; PA, periumbilical area; RLQ, right lower quadrant

**Figure 4:**
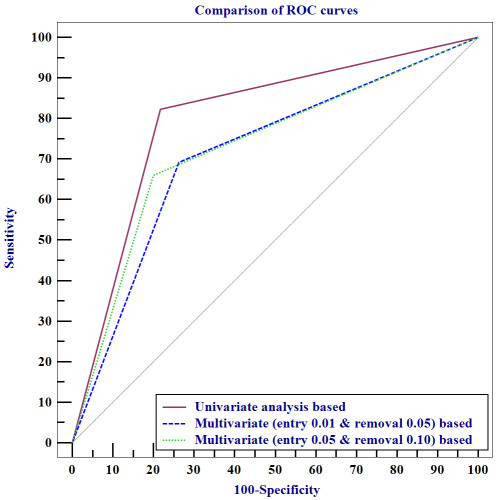
**Comparison of ROC curves for the decision support models**.

The six measures were compared using a 10-fold cross validation to assess the generalization ability of these decision support models. The differences in the clinical factors selected before and after the application of the C5.0 decision tree algorithm are shown in Tables 6 and 7. This showed that the decision support model based on univariate analysis was superior to those based on multivariate analyses with different conditions (Table 8). The decision support model based on the univariate analysis was statistically superior to those based on multivariate analyses in terms of predictive power and discriminatory capacity, which was expressed by the area under the ROC curve (*p *< 0.01, 95% CI, 3.13-14.5; *p *< 0.05, 95% CI, 1.54-13.1; Table 9 and Figure 4). The decision support model based on multivariate analysis using loose criteria was also better than that using strict criteria, especially the AUC measure, although the discriminatory power between the two models was not statistically significant (*p *= 0.400; 95% CI, -2.0-5.02).

**Table 6:** Clinical factors selected before the application of C5.0 decision tree algorithm during 10-fold cross validation

Variables	Fold 1			Fold 2			Fold 3			Fold 4			Fold 5			Fold 6			Fold 7			Fold 8			Fold 9			Fold 10		
	
	M1	M2	M3	M1	M2	M3	M1	M2	M3	M1	M2	M3	M1	M2	M3	M1	M2	M3	M1	M2	M3	M1	M2	M3	M1	M2	M3	M1	M2	M3
Age	O			O			O												O						O			O		
Complaints	O	O	O	O	O	O	O	O	O	O	O	O	O	O	O	O	O	O	O	O	O	O	O	O	O	O	O	O		
Urine pH																						O		O						
Urine glucose	O	O	O	O	O	O	O	O	O	O	O	O	O	O	O	O	O	O	O	O	O	O	O	O	O			O	O	O
Urine ketone	O			O			O			O			O			O			O			O			O			O		
Urine Urobilinogen	O			O			O																							
WBC	O			O			O		O	O		O	O			O			O			O		O	O			O		
RBC	O			O			O			O			O			O			O			O			O			O		
NEUT	O	O	O	O	O	O	O	O	O	O	O	O	O	O	O	O	O	O	O	O	O	O	O	O	O	O	O	O	O	O
LYMP	O			O			O			O			O			O			O			O			O			O		
MONO	O			O			O															O						O		
EOS	O			O			O			O			O			O			O			O		O	O			O		
BASO	O			O			O			O			O			O			O			O			O			O		
LUC	O			O			O			O			O			O			O			O			O			O		
Bilirubin (T)	O			O			O			O			O			O			O			O		O	O			O		
Bilirubin (D)	O			O			O		O	O			O			O	O	O	O			O			O			O		
Lipase	O	O	O	O			O	O	O	O			O	O	O	O			O			O	O	O	O			O	O	O
Amylase (T)							O									O														

M1: univariate analysis (*p *< 0.01)

M2: multivariate analysis (entry and removal criteria of 0.01 and 0.05)

M3: multivariate analysis (entry and removal criteria of 0.05 and 0.10)

**Table 7:** Clinical factors selected after the application of C5.0 decision tree algorithm during 10-fold cross validation

Variables	Fold 1			Fold 2			Fold 3			Fold 4			Fold 5			Fold 6			Fold 7			Fold 8			Fold 9			Fold 10		
	
	M1	M2	M3	M1	M2	M3	M1	M2	M3	M1	M2	M3	M1	M2	M3	M1	M2	M3	M1	M2	M3	M1	M2	M3	M1	M2	M3	M1	M2	M3
Age	O						O												O						O					
Complaints	O	O	O	O	O	O	O	O	O	O		O	O	O	O	O	O	O	O	O	O	O	O	O	O	O	O	O		
Urine pH																						O		O						
Urine glucose	O	O	O	O	O	O	O	O	O	O		O	O	O	O	O	O	O	O	O	O	O	O	O	O			O		
Urine ketone	O			O			O									O									O			O		
WBC	O			O			O		O			O	O			O			O			O		O	O			O		
RBC	O									O			O			O			O			O			O					
NEUT		O	O	O	O	O		O	O	O	O	O	O	O	O	O	O	O		O	O		O	O	O	O	O		O	O
LYMP	O			O			O			O			O			O			O			O			O			O		
MONO				O																										
EOS	O						O			O			O			O			O						O			O		
BASO	O			O			O			O			O			O			O			O			O			O		
LUC	O			O			O			O						O			O			O			O			O		
Bilirubin (T)	O			O			O			O			O			O			O			O			O			O		
Bilirubin (D)	O			O					O	O			O			O	O	O				O			O			O		
Lipase	O	O	O	O			O	O	O	O			O						O			O			O			O	O	O
Amylase (T)							O									O														

M1: C5.0 decision tree algorithm with univariate analysis (*p *< 0.01)

M2: C5.0 decision tree algorithm with multivariate analysis (entry and removal criteria of 0.01 and 0.05)

M3: C5.0 decision tree algorithm with multivariate analysis (entry and removal criteria of 0.05 and 0.10)

**Table 8:** Performance of decision support models based on univariate and multivariate analysis (10-fold cross validation)

Performance	ACC	SENS	SPEC	PPV	NPV	AUC
**Based on univariate** analysis*	80.2	82.4	78.3	76.8	83.5	80.3
**Based on multivariate** analysis**	71.6	69.3	73.7	69.7	73.3	71.5
**Based on multivariate** **analysis**^**†**^	73.5	66.0	80.0	74.3	72.9	73.0

* *p *< 0.01; standard error: 2.54 (95% CI, 75.6-84.5)

** entry and removal criteria of 0.01 and 0.05; standard error: 2.89 (95% CI, 66.3-76.3)

^† ^entry and removal criteria of 0.05 and 0.10; standard error: 2.86 (95% CI, 67.9-77.7)

**Table 9:** Discriminatory capacity of decision support models used for the diagnosis of acute appendicitis expressed as areas under ROC curves (95% CI)

Pairwise comparison of ROC curves	Based on univariate analysis*	Based on multivariate analysis**	**Based on multivariate analysis**^**†**^
Based on univariate analysis*	-	*p *< 0.01 (95% CI: 3.13-14.5)	*p *< 0.05 (95% CI, 1.54-13.1)
Based on multivariate analysis**	*p *< 0.01 (95% CI, 3.13-14.5)	-	*p *= 0.400 (95% CI, -2.0-5.02)
Based on multivariate analysis^†^	*p *< 0.05 (95% CI, 1.54-13.1)	*p *= 0.400 (95% CI, -2.0-5.02)	-

* *p *< 0.01

** entry and removal criteria of 0.01 and 0.05

^† ^entry and removal criteria of 0.05 and 0.10

## Discussion

From a clinical viewpoint, one of the most difficult problems is distinguishing patients with suspected acute appendicitis from those with acute abdominal pain. Thus, we developed a hybrid decision support model based on a decision tree algorithm and statistical analysis to reduce the high workload of clinicians. We also investigated the different diagnostic knowledge provided by the decision support models. We extracted subsets from the univariate analysis-based model (lymphocytes, urine glucose, total bilirubin, total amylase, chloride, red blood cell, neutrophils, eosinophils, white blood cell, complaints, basophils, glucose, monocytes, activated partial thromboplastin time, urine ketone, direct bilirubin) and from the multivariate analysis based model (neutrophils, complaints, total bilirubin, urine glucose, lipase) that were indispensable for discovering early diagnostic knowledge (i.e., relationships among these parameters) related to acute appendicitis, although several criteria did not reach statistical significance (Tables 4 and 5).

The clinical parameters included well-known risk factors for acute appendicitis described in previous studies, i.e., neutrophils (or lymphocytes), eosinophils, RLQ tenderness, amylase, and lipase. Kalan [26] produced a modified Alvarado score by removing neutrophils from the model. However, the present study showed that the neutrophil count is a very important factor when evaluating patients with acute appendicitis [27], especially children [28,29]. Clark [30] tested the eosinophil count in the diagnostic evaluation of patients presenting with acute abdominal pain who subsequently underwent appendectomy and whether eosinophilia was related to subsequent histology. Patients with abdominal pain and peripheral eosinophils appeared less likely to have acute appendicitis based on their subsequent histology. Santosh [31] reported significant local eosinophil activation and degranulation during acute appendicitis, which was sufficient to elevate serum levels of eosinophil chemotactic protein. However, the inverse relationship between the duration of symptoms and serum eosinophil cationic protein was not statistically significant in cases of acute appendicitis. Um [32] reported the case of a 17-year-old female, who was characterized by increased serum amylase activities combined with normal serum lipase, normal creatinine, and a low amylase/creatinine clearance ratio. She was diagnosed with macroamylasemia and acute appendicitis without apparent clinical symptoms of a pancreatic disorder.

We performed a 10-fold cross validation to estimate the diagnostic accuracy of the decision support models. The results showed that the larger induced decision model was more effective at distinguishing patients with acute appendicitis from those with acute abdominal pain, whereas the compact decision model, with a smaller induced decision tree, was less accurate for the test data. The range of diagnostic accuracy was approximately 83-94% for the full dataset and 72-80% for the 10-fold cross validation, compared with the average accuracy in previous studies of acute abdominal pain [14,15] or acute appendicitis [5,16]. Several explanations provided similar or better results, which can be summarized as follows.

I) In our dataset, the sample size of each decision or diagnosis had a balanced distribution. In contrast, the distribution of diagnoses reported for these studies was extremely imbalanced, e.g., one diagnosis was represented by a significantly lower number of cases than the others [15]. The decision boundary learned by a standard machine learning algorithm, such as a decision tree algorithm, can be severely skewed toward either a positive or negative decision. Consequently, the false negative or positive rate can be excessively high. One research approach for overcoming the class imbalance problem is to resample the original training dataset, by either oversampling the minority class and/or undersampling the majority class until decisions are represented in a more balanced way [33].

II) We used the reduced clinical parameters set after applying the univariate or multivariate analysis as a feature selection approach when constructing the final decision support model. This dataset was smaller compared with the number of parameters used in previous studies. Our dataset quality may even be increased by selecting informative features from a high-dimensional dataset. This reduces the time required to perform induction and it makes the resulting rules more comprehensible, thereby increasing the resulting accuracy [34,35].

This study had the following limitations because of its retrospective study design. The number of patients with acute appendicitis and non-acute appendicitis was relatively small, which produced variations when deriving the relevant parameters and their relationships. The feasibility of using derived rules has been verified using an external validation study [4] or a prospective studies [9,15,16]. These considerations may provide fruitful directions for further research.

## Conclusions

This study developed a simple and reliable hybrid decision support model based on statistical analyses and a decision tree algorithm to provide high accuracy, early diagnosis of patients with suspected acute appendicitis. This model also facilitated diagnostic knowledge discovery using the derived rules. The experimental results show that a decision support model based on univariate analysis provided excellent discrimination and we demonstrated its feasibility for predicting acute appendicitis. Therefore, the decision model developed in our study can be applied to support the initial decision of clinicians and increase vigilance when detecting suspected acute appendicitis.

## Abbreviations

S.G: Specific gravity; O.B: Occult blood; WBC: White blood cell; RBC: Red blood cell; Ep. Cell: Epithelial cell: HGB: Hemoglobin; HCT: Hematocrit; MCV: Mean corpuscular volume; MCH: Mean corpuscular hemoglobin; MCHC: Mean corpuscular hemoglobin concentration; PLT: Platelet count; NEUT: Neutrophils; LYMP: Lymphocytes; MONO: Monocytes; EOS: Eosinophils; BASO: Basophils; LUC: Large unstained cells; MPV: Mean platelet volume; Na: Sodium; K: Potassium; Cl: Chloride; BUN: Blood urea nitrogen; ALP: Alkaline phosphatase; AST: Aspartate aminotransferase; ALT: Alanine aminotransferase; APTT: Activated partial thromboplastin time; PT: Prothrombin time.

## Competing interests

The authors declare that they have no competing interests.

## Authors' contributions

CSS conceived the study and participated in data analysis and the computation of performance values. BKJ was the consultant for the knowledge of (differential) diagnosis of acute appendicitis disease. STS and MSK interpreted the concept of the model prototype and helped revise the manuscript. YNK is the corresponding author who conceived the study and drafted the manuscript. All authors read and approved the final manuscript.

## Pre-publication history

The pre-publication history for this paper can be accessed here:

http://www.biomedcentral.com/1472-6947/12/17/prepub
